# Pentoxifylline and Methylprednisolone Additively Alleviate Kidney Failure and Prolong Survival of Rats after Renal Warm Ischemia-Reperfusion

**DOI:** 10.3390/ijms19010221

**Published:** 2018-01-11

**Authors:** Grzegorz Wystrychowski, Wojciech Wystrychowski, Władysław Grzeszczak, Andrzej Więcek, Robert Król, Antoni Wystrychowski

**Affiliations:** 1 Department of Internal Medicine, Diabetology and Nephrology, School of Medicine with the Division of Dentistry in Zabrze, Medical University of Silesia in Katowice, 41-800 Zabrze, Poland; wgrzeszczak@sum.edu.pl; 2 Department of General, Vascular and Transplant Surgery, School of Medicine in Katowice, Medical University of Silesia in Katowice, 40-027 Katowice, Poland; wwystrych@gmail.com (W.W.); robertk@hot.pl (R.K.); 3 Department of Nephrology, Transplantation and Internal Medicine, School of Medicine in Katowice, Medical University of Silesia in Katowice, 40-027 Katowice, Poland; awiecek@spskm.katowice.pl (A.Wi.); awystrych@gmail.com (A.Wy.)

**Keywords:** ischemia, reperfusion, ischemia-reperfusion injury, acute kidney injury, chronic kidney disease, pentoxifylline, steroid, survival, mortality, kidney transplantation

## Abstract

Renal ischemia-reperfusion injury (IRI) induces local inflammation leading to kidney damage. Since pentoxifylline (PTX) and steroids have distinct immunomodulatory properties, we aimed to evaluate for the first time their combined use in IRI-induced acute kidney injury (AKI) and chronic kidney disease (CKD) in rats. In two experiments, PTX (100 mg/kg body weight subcutaneously) was administered 90 min prior to renal IRI or/and methylprednisolone (MP; 100 mg/kg body weight intramuscularly) was infused 60 min after reperfusion of a solitary kidney (AKI model: 45 min ischemia, 48 male Sprague-Dawley rats) or one kidney with excision of contralateral kidney 2 weeks later (CKD model: 90 min ischemia, 38 rats). Saline was infused in place of PTX or/and MP depending on the group. Renal function (diuresis, serum creatinine, creatinine clearance, sodium and potassium excretion, and urine protein/creatinine) was assessed at 48 h and 120 h post-IRI (AKI model) or 4, 16 and 24 weeks after IRI, along with survival analysis (CKD model). More evidently at early stages of AKI or CKD, treated animals showed higher glomerular filtration and diminished tubular loss of electrolytes, more so with PTX + MP than PTX or MP (serum creatinine (μmol/L) at 48 h of AKI: 60.9 ± 19.1 vs. 131.1 ± 94.4 vs. 233.4 ± 137.0, respectively, vs. 451.5 ± 114.4 in controls, all *p* < 0.05; and at 4 weeks of CKD: 89.0 ± 31.9 vs. 118.1 ± 64.5 vs. 156.9 ± 72.6, respectively, vs. 222.9 ± 91.4 in controls, *p* < 0.05 for PTX or PTX + MP vs. controls and PTX + MP vs. MP). Survival was better by >2-fold with PTX + MP (89%) vs. controls (40%; *p* < 0.05). PTX + MP largely protect from IRI-induced AKI and CKD and subsequent mortality in rats. This calls for clinical investigations, especially in kidney transplantation.

## 1. Introduction

Renal ischemia-reperfusion injury (IRI) is a common complication of renal hypoperfusion in the clinical settings of shock, renal vascular occlusion, hepatorenal syndrome, or kidney transplantation. IRI involves glomerular capillary endothelial dysfunction and tubular epithelial necrosis induced by ischemia, as well as reactive oxygen species generation during reperfusion, both initiating a robust inflammatory reaction [1]. In consequence, progressive glomerular sclerosis and tubular atrophy with interstitial fibrosis may develop [2].

Kidney transplantation involves IRI to the graft due to warm and cold ischemia at organ procurement and storage, respectively, and reperfusion at implantation. Clinical outcome analyses indicate IRI as the crucial factor affecting long-term kidney allograft function [3]. Furthermore, high rates of delayed graft function (20–25% [4]) prove that standard glucocorticosteroid-based immunosuppressive therapy at and following kidney transplantation does not provide sufficient attenuation of inflammatory reaction triggered by IRI, nor do current practices of kidney donor management or graft preservation. Despite some experimental data showing favorable effects of steroid administration in renal IRI (protection of tubular epithelium morphology [5], reduced kidney leukocyte infiltration, plasma interleukin (IL)-6 and serum creatinine concentrations [6]) the clinical studies were disappointing. The largest trial conducted so far (Steroids In caRdiac Surgery) failed to show any nephroprotective effect of intraoperative double 250 mg methylprednisolone (MP) i.v. administration in the 30 days following cardiopulmonary bypass operations in >7500 patients [7].

Numerous experimental studies demonstrated protective effects of a non-selective phosphodiesterase inhibitor pentoxifylline (PTX) applied before organ ischemia or transplantation, attributable to its unique anti-inflammatory properties. This protection against IRI has been shown for the most part in renal, intestinal, and liver ischemia-reperfusion animal models [8,9,10]. On the other hand, sparse clinical trials showed that long-term use of PTX reduces albuminuria or glomerular filtration loss in diabetic and non-diabetic kidney disease [11,12]. Given these promising outcomes, we aimed to assess for the first time how the combined use of PTX and a steroid (MP) in the peri-ischemic period affects the course of IRI-induced acute kidney injury, as well as the progression to chronic kidney disease and long-term survival in the rat model.

## 2. Results

All studied animals survived infusions, anesthesia and surgical procedures.

### 2.1. Acute Kidney Injury Experiment

At 48 h after IRI of the solitary kidney, the control rats that were not administered any of the studied active compounds (Group 1) exhibited signs of severe kidney failure with preserved diuresis, yet highly elevated urinary excretion of sodium and potassium (Table 1), as related to intact or uninephrectomized non-IRI rats (Table 2). On the other hand, the animals that were treated with PTX or MP (Groups 2 and 3, respectively) had remarkably improved glomerular filtration (in terms of both serum creatinine and creatinine clearance) and diminished tubular loss of both electrolytes, these beneficial effects being more pronounced with PTX than MP administration. Furthermore, the simultaneous use of both PTX and MP resulted in the greatest alleviation of the kidney failure—rats of Group 4 had the serum creatinine levels comparable to uninephrectomized rats that did not undergo renal IRI (Group UNX; *p* = 0.38), the highest creatinine clearance, and ≥20-fold lower urinary loss of sodium and potassium than controls, equivalent to that in Group UNX (*p* = 0.69 and *p* = 0.39, respectively). Of note, there were no significant differences in proteinuria between the experimental groups, which was several times higher than in non-IRI rats.

**Figure 1:**
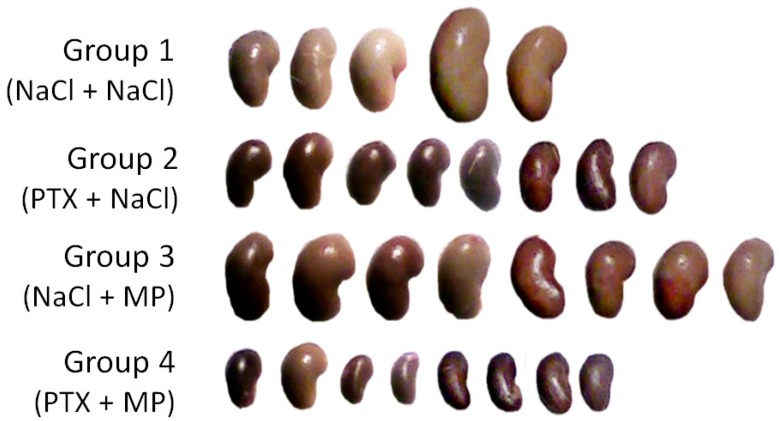
Left (solitary) kidneys excised at 120 h after the ischemia-reperfusion injury (IRI): in the control rats administered saline infusions (Group 1), rats administered pentoxifylline (PTX) prior to IRI (Group 2), those administered methylprednisolone (MP) post IRI (Group 3) and those infused with both PTX and MP around IRI (Group 4) (photographs of the majority of excised organs).

During the following three days, 4 rats of Group 1, 1 rat of Group 2 and 1 of Group 3, and no rats of Group 4 died. At 120 h post-IRI, renal function was largely restored in the controls that remained alive, and matched that of animals treated with MP in terms of glomerular filtration and electrolyte excretion (Table 1). It was, nevertheless, still inferior to kidney performance in rats that were administered PTX or PTX and MP with regard to serum creatinine, creatinine clearance (marginal statistical significance) or potassium excretion (Table 1), in whom these parameters matched the levels in the intact rats of Group 0 (all *p* > 0.05, Table 2). At this stage, the control animals also displayed higher urine volume, whereas rats treated with the combination of PTX and MP showed higher proteinuria than all other groups. In addition, left (solitary) kidney weight was significantly lower in the animals that were administered PTX (Table 1 and Figure 1), and with combined use of PTX and MP it was not different from that in both non-IRI groups (*p* = 0.83 and *p* = 0.23, respectively, Table 2).

### 2.2. Chronic Kidney Disease Experiment

**Figure 2:**
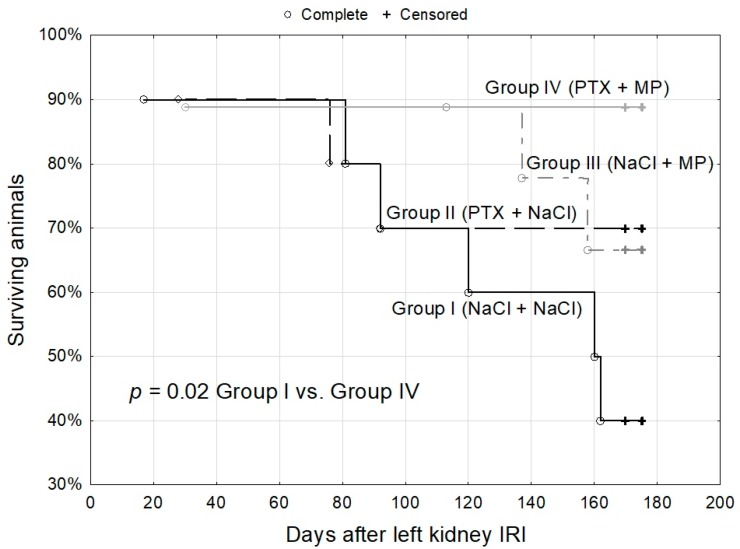
Chronic kidney disease model—survival of the animals in the 24-week observation after the prolonged ischemia of left kidney with subsequent excision of right kidney at 2 weeks after left kidney IRI: in the control rats administered saline infusions (Group I), rats administered PTX prior to IRI (Group II), those administered MP post IRI (Group III) and those infused with both PTX and MP around IRI (Group IV). Kaplan-Meier curves; log-rank test.

The weight of the right kidney excised 2 weeks after prolonged ischemia of the left kidney was lower in the two groups that were administered MP (Table 3). Two weeks later, despite comparable urine output, glomerular filtration and electrolyte reabsorption were less impaired in the groups administered PTX and/or MP, with the greatest benefit of their combined use (~2.5-fold higher glomerular filtration, ~10-fold lower sodium loss, ~3.5-fold lower potassium loss as compared to controls). During the 20 weeks of further observation survival of the animals was superior in Group IV (~90%), as compared with Groups II and III (~70%) or the controls of Group I (statistically significantly—40%) (Figure 2). At 16 and 24 weeks post-IRI, the studied renal function parameters still differed noticeably between the living animals of the experimental groups in the same pattern as at week 4, albeit with lesser statistical significances, and remained somewhat inferior in PTX and/or MP-treated animals than in non-IRI groups (all *p* < 0.05, Table 2 and Table 3). On the other hand, proteinuria uniformly increased over time across all four groups and was ~5–7-fold higher than in non-IRI uninephrectomized rats (Group UNX) and ~10–20-fold higher than in intact animals (all *p* < 0.05).

## 3. Discussion

The presented results indicate that PTX and MP complement each other in their nephroprotective effects in the settings of IRI-induced acute kidney injury or chronic kidney disease. With combined use of PTX and MP, both glomerular filtration and tubular absorptive capacities appeared to be largely protected from the deleterious sequelae of warm renal ischemia and subsequent reperfusion, which translated into enhanced long-term survival of the animals. The extents of renal function improvements with MP, PTX, and MP + PTX speak in favor of an additive, rather than synergistic, nephroprotective effect of the drug combination. Notably, the apparent loss of much of these gains at later stages of both experiments, as compared to controls, seems to be merely a consequence of higher mortality in the latter group and availability of only the least affected control animals for the follow-up statistical comparisons. Eventually, although the study lacks insight into the biological mechanisms of the revealed effects, the apparent reduction in renal edema, knowledge of the actions of PTX or steroids at the molecular level, as well as findings from their applications in IRI by others, point to the complementary immunomodulating effects of MP and PTX.

In the experimental settings of inflammation PTX, by increasing intracellular cyclic adenosine monophosphate (cAMP) (or in less elucidated cAMP-independent ways), reduces expression of pro-inflammatory chemokines, cytokines (*tumor necrosis factor-α* (*TNFα*), in particular) and adhesion molecules [13,14], which attenuates activation of macrophages [15], impairs adhesion and activation of dendritic cells [16] and T lymphocytes [17], and diminishes neutrophil oxidative burst [18]. Furthermore, by inhibiting synthesis of transforming growth factor-β and connective tissue growth factor, PTX diminishes collagen synthesis in vitro [19,20] and renal interstitial fibrosis in the rat model of obstructive nephropathy [21].

On the other hand, glucocorticosteroids act primarily via binding to the steroid receptor—they regulate its activity as a nuclear transcription factor, influencing promoter sequences and gene expression (genomic mechanism). In particular, they thus inhibit transcription of *inducible nitric oxide synthase*, *selectins and adhesion molecules*, as well as neutralize transcription factors activator protein-1 and nuclear factor κ-light-chain-enhancer of activated B cells (NFκB). At the post-transcriptional level, steroids destabilize mRNA for inflammatory mediators (TNFα, IL-6, IL-1β) [22]. Finally, in a non-genomic mechanism, they exert anti-apoptotic effects (Bcl up- and Bax down-regulation) in human proximal kidney tubule cells [5]. 

PTX and steroids share some immunomodulating actions (like inhibition of IL-2 receptor expression, IL-2 and interferon-γ release), yet the corresponding biological mechanisms appear different [23]. For example, both compounds downregulate expression of *TNFα*. However, as shown by Han et al. in their pioneer study, while steroids (dexamethasone) inhibit the lipopolysaccharide-induced synthesis of TNFα in macrophages more robustly at the translational than at the transcriptional level, PTX selectively diminishes this cytokine mRNA synthesis [24]. Recent studies in the rat models of ischemic brain injury or angiotensin II-induced cardiac hypertrophy show that this effect of PTX is due to the inhibition of toll-like receptor 4/NFκB pathway activity [25,26].

In view of the expected synergism, the combination of steroid and PTX has been studied in a few experimental studies. In vitro, PTX enabled a decrease in steroid dose necessary to inhibit the induced inflammatory cytokine expression in peripheral blood mononuclear cells [23]. In another study, added to dexamethasone, it enhanced the alleviation of an induced limb ischemia in rat by 2-fold [27]. In the rat model of biliary obstruction, treatment with PTX or/and prednisolone for 30 days resulted in reduced portal fibrosis, however with no additive effect of the drug combination [28]. The clinical setting where the effects of PTX were repeatedly confronted with those of steroids was alcoholic hepatitis. However, the comparative studies of a relatively short application of the compounds (28–30 days) were inconsistent as to survival benefit [29,30], and the only trial that evaluated a 1-month concomitant use of PTX and steroid (prednisolone) did not show any improvement in the 6-month survival of 270 patients, compared to steroid therapy only. Nevertheless, the drug combination decreased the rates of hepatorenal syndrome almost 2-fold with marginal statistical significance (*p* = 0.07) [31].

Curiously, neither MP, nor PTX, nor their concomitant use alleviated proteinuria in our study. On the contrary, with the combined treatment proteinuria was ~2-fold higher on the 5th day of acute kidney injury (Table 1). However, the accompanying remarkably high glomerular filtration with low serum creatinine concentration indicates that proteinuria was transient and resultant from glomerular hyperfiltration typical of an early phase of post-IRI kidney regeneration.

In final remarks, addressing the rationale of the applied methodology, it has to be noted that the applied dose of PTX was within the range of those used in other experiments on rats: 100 mg/kg body weight vs. 30–300 mg/kg body weight [21,25,27], and its application at this dose prior to IRI was previously found by us to be nephroprotective, as opposed to intra- or post-IRI administration [10]. On the other hand, the dosage of MP (100 mg/kg body weight) was higher than that usually studied in IRI rat models (30 mg/kg body weight) [32,33]. This more intense steroid treatment was based on our previous experiments with MP doses ranging from 15 to 100 mg/kg body weight, which showed the long-term kidney protection from IRI only with the highest dosage [34].

Eventually, our results implicate usefulness of PTX and steroid combination in the clinical settings of renal IRI, with PTX treatment initiation preceding the occurrence of IRI. This points to the circumstances of a predictable renal IRI, like kidney transplantation or major surgeries, as the most appropriate for the future clinical studies. Potential benefits of PTX in peritransplant period are to be expected with donor pretreatment in particular. The additive immunomodulating actions of PTX and steroids may necessitate stronger antibiotic coverage, but should allow steroid dosage reduction and consequently reduce the risk of their long-term adverse effects.

## 4. Materials and Methods

Two experiments were carried out.

**Figure 3:**
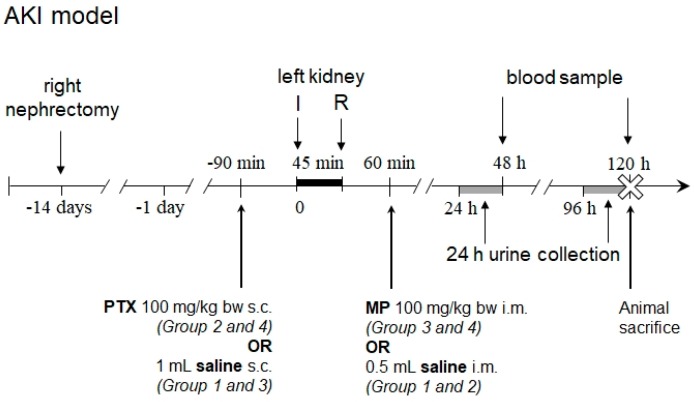
Scheme of the experiment of renal ischemia-reperfusion injury-induced acute kidney injury (AKI). I—onset of ischemia; R—onset of reperfusion; PTX—pentoxifylline; MP—methylprednisolone; bw—body weight; s.c.—subcutaneously; i.m.—intramuscularly.

In order to assess the influence of PTX and glucocorticosteroids on ischemic acute renal failure, 14 days after right nephrectomy, renal in situ IRI was induced in 48 (4 groups of 12) male 9-week old Sprague-Dawley rats by 45 min clamping of left renal vascular pedicle. PTX (Polfa, Warsaw, Poland) 100 mg/kg body weight in 1 mL 0.9% NaCl (Group 2 and 4) or 1 mL saline (Group 1 and 3) was injected subcutaneously 90 min before ischemia. Methylprednisolone (MP; Polfa) 100 mg/kg body weight (Group 3 and 4) or 0.5 mL saline (Group 1 and 2) was administered intramuscularly 60 min after reperfusion onset. Creatinine clearance, fractional excretions of sodium and potassium, and urine protein to creatinine ratio were estimated 48 h and 120 h after IRI (Figure 3).

**Figure 4:**
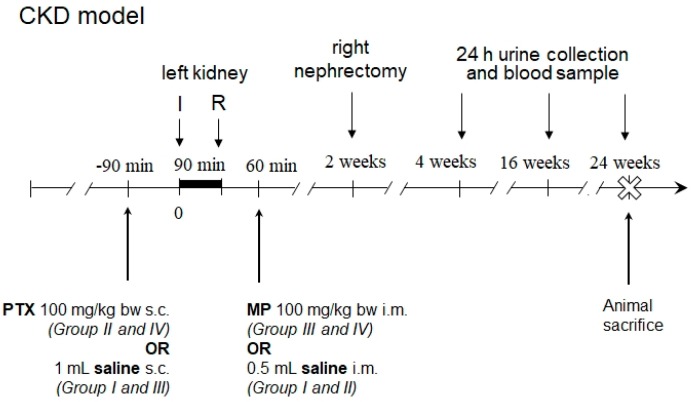
Scheme of the experiment of renal ischemia-reperfusion injury-induced chronic kidney disease (CKD). I—onset of ischemia; R—onset of reperfusion; PTX—pentoxifylline; MP—methylprednisolone.

For the evaluation of the effects of the studied compounds on the course of IRI-induced chronic kidney disease, rats underwent a prolonged 90 min-long warm ischemia of left kidney with preservation of intact right kidney to aid survival in the early post-ischemic period. 38 male 9-week old Sprague-Dawley rats were randomized into 4 groups. 90 min before left kidney ischemia, rats of Groups II (*n* = 9) and IV (*n* = 9) were given PTX 100 mg/kg body weight in 1 mL 0.9% NaCl subcutaneously and rats of Groups I (*n* = 10) and III (*n* = 10) 1 mL saline only. After 60 min of reperfusion Groups III and IV were given MP (100 mg/kg body weight in 0.5 mL 0.9% NaCl intramuscularly) and Groups I and II 0.9% NaCl only. Two weeks after left kidney IRI induction, right nephrectomy was performed. Four, 16 and 24 weeks after IRI the above listed renal function parameters were measured. In addition, right and left kidney weights were measured (Figure 4).

Finally, renal function parameters in both experiments were confronted with those measured in our other experiments in rats that did not undergo renal IRI—nine intact 9-week old male Sprague-Dawley rats (Group 0), as well as sixteen 11-week old rats that were uninephrectomized 2 weeks earlier (Group UNX).

The animals were obtained from the Experimental Medicine Centre, Medical University of Silesia, Katowice, Poland. All rats were provided with standard laboratory chow and water ad libitum in a temperature-controlled environment (23 °C) with 12 h light-dark cycle. The surgical procedures were performed under ether anesthesia. During ischemia periods the animals were awaken. Prior to blood collections from retro-orbital plexus rats were kept for 24 h in metabolic cages with chow deprivation and continuous urine collection.

Serum creatinine concentration was estimated with a spectrophotometer (PZ Cormay S.A., Łomianki, Poland), plasma and urine electrolytes with a flame photometer (Eppendorf, Hamburg, Germany), urine creatinine concentration with a colorimetric picric acid method spectrophotometer (Karl Zeiss, Jena, Germany), and urine protein concentration with trichloroacetic acid precipitation method.

Statistical comparisons between the studied groups within each experiment were carried out with use of Kruskal-Wallis analysis of variance (ANOVA) and Mann-Whitney *U* test as *post-hoc*. Survival of animals during the 24 weeks of the second experiment was compared between the groups by Kaplan-Meier analysis and log-rank test. *p* < 0.05 was regarded as indicative of statistical significance.

The study protocol was approved by the local ethical committee for the experiments on animals (decision 64/2008, 14 October 2008).

## 5. Conclusions

The study shows for the first time additive nephroprotective effects of PTX and a steroid in experimental renal IRI, manifested in alleviation of induced acute kidney injury or chronic kidney disease, and improved survival. Clinical settings of inevitable or anticipated renal IRI, like kidney transplantation or major surgeries, shall benefit from this drug combination and call for relevant prospective trials.

## Figures and Tables

**Table 1:** Acute kidney injury model—renal function parameters at 48 and 120 h after the ischemia-reperfusion of the solitary kidney.

Clinical Parameters	Group 1 NaCl + NaCl	Group 2 PTX + NaCl	Group 3 NaCl + MP	Group 4 PTX + MP
**48 h**	*n* = 12	*n* = 12	*n* = 12	*n* = 12
Body weight (g)	349 ± 32	341 ± 19	338 ± 23	330 ± 28
Diuresis (mL)	17.5 ± 10.5	21.7 ± 7.0	31.2 ± 9.7 *^,^^	20.9 ± 13.1 ^#^
Serum creatinine (µmol/L)	451.5 ± 114.4	131.1 ± 94.4 *	233.4 ± 137.0 *^,^^	60.9 ± 19.1 *^,^^^,#^
Creatinine clearance (mL/min/kg body weight)	0.11 ± 0.16	1.47 ± 0.93 *	0.84 ± 0.71 *	2.21 ± 0.86 *^,#^
Fractional excretion of sodium (%)	23.1 ± 20.7	0.84 ± 0.78 *	2.59 ± 2.40 *^,^^	0.38 ± 0.22 *^,#^
Fractional excretion of potassium (%)	717 ± 323	80.2 ± 60.1 *	237 ± 223 *	36.8 ± 14.5 *^,^^^,#^
Urine protein/urine creatinine (g/g)	13.1 ± 7.6	3.0 ± 4.8	9.0 ± 7.1	7.1 ± 4.4
**120 h**	*n* = 8	*n* = 11	*n* = 11	*n* = 12
Body weight (g)	319 ± 21	335 ± 19	299 ± 26 ^	319 ± 29
Diuresis (mL)	32.2 ± 7.6	16.1 ± 5.4 *	21.9 ± 15.1 *	11.7 ± 3.9 *^,^^^,#^
Serum creatinine (µmol/L)	98.9 ± 44.2	47.6 ± 6.7 *	82.1 ± 55.2	39.5 ± 9.8 *^,^^^,#^
Creatinine clearance (mL/min/kg body weight)	2.00 ± 1.23	3.21 ± 1.17 ^$^	2.46 ± 1.17	3.70 ± 1.96 ^$^
Fractional excretion of sodium (%)	0.31 ± 0.22	0.17 ± 0.06	0.24 ± 0.25	0.14 ± 0.12
Fractional excretion of potassium (%)	90.7 ± 48.8	34.2 ± 14.8 *	75.8 ± 82.7	36.4 ± 31.8 *
Urine protein/urine creatinine (g/g)	1.30 ± 0.56	1.17 ± 0.66	0.98 ± 0.35	2.50 ± 4.58 *^,^^^,#^
Left kidney weight (g)	3.26 ± 0.71	1.79 ± 0.34 *	2.80 ± 0.52 ^	1.53 ± 0.45 *^,^^^,#^

Group 1—control rats administered saline infusions before and after renal ischemia-reperfusion injury (IRI); Group 2—rats administered pentoxifylline (PTX) prior to IRI; Group 3—rats administered methylprednisolone (MP) post IRI; Group 4—animals infused PTX before IRI and MP post IRI; Means ± standard deviations; * *p* < 0.05 vs. Group 1; ^ *p* < 0.05 vs. Group 2; ^#^ *p* < 0.05 vs. Group 3; Kruskal-Wallis ANOVA + Mann-Whitney *U* test; ^$^ *p* = 0.07 vs. Group 1; Mann-Whitney *U* test.

**Table 2:** Renal function parameters in intact 9-week-old male Sprague-Dawley rats (Group 0) and in 11-week-old such rats at 2 weeks after unilateral nephrectomy (Group UNX).

Clinical Parameters	Group 0 *n* = 9	Group UNX *n* = 16
Body weight (g)	298 ± 12	347 ± 33 *
Diuresis (mL)	12.5 ± 3.9	17.1 ± 4.2 *
Serum creatinine (µmol/L)	44.2 ± 4.2	51.9 ± 6.1 *
Creatinine clearance (mL/min/kg body weight)	3.62 ± 0.72	3.14 ± 0.64 ^
Fractional excretion of sodium (%)	0.15 ± 0.04	0.37 ± 0.13 *
Fractional excretion of potassium (%)	22.0 ± 3.65	32.7 ± 8.32 *
Urine protein/urine creatinine (g/g)	0.80 ± 0.26	1.74 ± 0.69 *
Left kidney weight (g)	1.40 ± 0.08	1.62 ± 0.21 *

Means ± standard deviations; * *p* < 0.05, ^ *p* = 0.09 vs. Group 0; Mann-Whitney *U* test.

**Table 3:** Chronic kidney disease model—renal function parameters at 4, 16 and 24 weeks after the prolonged ischemia of left kidney with subsequent excision of right kidney at 2 weeks after left kidney ischemia-reperfusion.

Clinical Parameters	Group I NaCl + NaCl	Group II PTX + NaCl	Group III NaCl + MP	Group IV PTX + MP
**2 weeks**				
Right kidney weight (g)	1.84 ± 0.19	1.75 ± 0.26	1.68 ± 0.11 *^,^^	1.47 ± 0.23 *^,^^^,#^
**4 weeks**	*n* = 10	*n* = 9	*n* = 10	*n* = 9
Body weight (g)	311 ± 41	313 ± 42	322 ± 22	308 ± 29
Diuresis (mL)	33.3 ± 9.4	26.6 ± 10.3	27.5 ± 7.8	22.3 ± 7.9
Serum creatinine (µmol/L)	222.9 ± 91.4	118.1 ± 64.5 *	156.9 ± 72.6	89.0 ± 31.9 *^,#^
Creatinine clearance (mL/min/kg body weight)	0.77 ± 0.44	1.85 ± 0.89 *	1.39 ± 0.87 *	1.90 ± 0.67 *^,#^
Fractional excretion of sodium (%)	2.17 ± 2.29	0.36 ± 0.56 *	0.42 ± 0.36 *^,^^	0.24 ± 0.11 *^,#^
Fractional excretion of potassium (%)	206.9 ± 120.6	73.7 ± 41.8 *	123.8 ± 83.1 *	61.4 ± 25.4 *^,^^^,#^
Urine protein/urine creatinine (g/g)	2.89 ± 1.83	2.57 ± 0.99	2.78 ± 1.65	2.60 ± 1.58
**16 weeks**	*n* = 6	*n* = 8	*n* = 7	*n* = 8
Body weight (g)	450 ± 63	483 ± 28	506 ± 32	513 ± 66
Diuresis (mL)	26.7 ± 4.1	22.5 ± 7.9	20.7 ± 5.9	18.7 ± 8.2
Serum creatinine (µmol/L)	150.0 ± 59.8	94.6 ± 58.6	88.3 ± 28.8 *	63.6 ± 25.8 *
Creatinine clearance (mL/min/kg body weight)	0.81 ± 0.55	2.07 ± 1.16 *	1.49 ± 0.81	2.15 ± 1.26 *
Fractional excretion of sodium (%)	0.29 ± 0.23	0.21 ± 0.26	0.77 ± 1.51	0.34 ± 0.56
Fractional excretion of potassium (%)	135.4 ± 89.6	47.9 ± 30.6 *	66.1 ± 76.8	58.2 ± 63.4
Urine protein/urine creatinine (g/g)	13.7 ± 9.0	7.6 ± 5.2	9.5 ± 9.4	8.6 ± 5.9
**24 weeks**	*n* = 4	*n* = 6	*n* = 7	*n* = 8
Body weight (g)	424 ± 120	515 ± 35	523 ± 29	544 ± 63
Diuresis (mL)	23.1 ± 4.6	22.8 ± 9.3	27.1 ± 12.2	20.4 ± 8.3
Serum creatinine (µmol/L)	216.0 ± 165.1	66.5 ± 46.4	86.3 ± 33.7	66.1 ± 39.3 ^$^
Creatinine clearance (mL/min/kg body weight)	1.08 ± 1.14	2.48 ± 1.99	1.75 ± 1.04	2.44 ± 1.41
Fractional excretion of sodium (%)	0.54 ± 0.59	0.25 ± 0.22	0.19 ± 0.13	0.15 ± 0.12
Fractional excretion of potassium (%)	139.9 ± 146.9	43.2 ± 46.4	56.7 ± 31.0	43.4 ± 34.2
Urine protein/urine creatinine (g/g)	18.3 ± 13.3	14.7 ± 13.6	11.0 ± 6.8	10.1 ± 5.1
Left kidney weight (g)	2.23 ± 0.76	2.29 ± 0.44	2.42 ± 0.29	2.67 ± 0.71

Group 1—control rats administered saline infusions before and after left kidney IRI; Group 2—rats administered PTX prior to IRI; Group 3—rats administered MP post IRI; Group 4—animals infused PTX before IRI and MP post IRI; Means ± standard deviations; * *p* < 0.05 vs. Group I; ^ *p* < 0.05 vs. Group II; ^#^ *p* < 0.05 vs. Group III; Kruskal-Wallis ANOVA + Mann-Whitney *U* test; ^$^ *p* = 0.07 vs. Group I; Mann-Whitney *U* test.

## Abbreviations

IRI	Ischemia-Reperfusion Injury
AKI	Acute Kidney Injury
ANOVA	Analysis of Variance
cAMP	Cyclic Adenosine Monophosphate
CKD	Chronic Kidney Disease
IL	Interleukin
MP	Methylprednisolone
NaCl	Sodium Chloride
NFκB	Nuclear Factor κ-light-chain-enhancer of activated B cells
PTX	Pentoxifylline
TNFα	Tumor Necrosis Factor-α
